# Generating Adaptive Behaviour within a Memory-Prediction Framework

**DOI:** 10.1371/journal.pone.0029264

**Published:** 2012-01-17

**Authors:** David Rawlinson, Gideon Kowadlo

**Affiliations:** 1 Department of Electrical and Electronic Engineering, National ICT Australia, University of Melbourne, Victoria, Australia; 2 Outware Mobile Pty. Ltd., Melbourne, Victoria, Australia; University of Sheffield, United Kingdom

## Abstract

The Memory-Prediction Framework (MPF) and its Hierarchical-Temporal Memory implementation (HTM) have been widely applied to unsupervised learning problems, for both classification and prediction. To date, there has been no attempt to incorporate MPF/HTM in reinforcement learning or other adaptive systems; that is, to use knowledge embodied within the hierarchy to control a system, or to generate behaviour for an agent. This problem is interesting because the human neocortex is believed to play a vital role in the generation of behaviour, and the MPF is a model of the human neocortex.

We propose some simple and biologically-plausible enhancements to the Memory-Prediction Framework. These cause it to explore and interact with an external world, while trying to maximize a continuous, time-varying reward function. All behaviour is generated and controlled within the MPF hierarchy. The hierarchy develops from a random initial configuration by interaction with the world and reinforcement learning only. Among other demonstrations, we show that a 2-node hierarchy can learn to successfully play “rocks, paper, scissors” against a predictable opponent.

## Introduction

### Adaptive Agents and Reinforcement Learning

In Artificial Intelligence, an adaptive intelligent agent is an entity (generally, a software program) that continuously learns to interact with a real or virtual world in such a way that it increasingly satisfies some internal objectives or optimization criteria [1]. By this definition, people could be classed as adaptive intelligent agents because we try to minimize pain and hunger, and maximize personal comfort.

Agents generally learn to behave in an adaptive way by interacting with an external world and discovering the consequences of actions. Learning algorithms can be placed into three classes depending on the type of feedback given during learning. “Unsupervised” methods learn patterns in data, without guidance or preference for particular patterns. “Supervised” learning requires ideal output values to be provided for each set of given inputs. During learning, a supervised algorithm adjusts its output to match the ideal values provided. After learning, it is hoped that the supervised system can generalize beyond its training data and produce good outputs from unseen inputs. A classic example of a supervised-learning artificial neural network is the multi-layer perceptron [2].

A third class of algorithms uses a technique called “reinforcement learning”, that requires an objective measure of output or world-state quality, called “reward” [3], [4]. The objective of reinforcement learning is to learn behaviour that maximizes cumulative reward. Reinforcement learning is often possible in situations where the ideal output is unknown or difficult to compute. For example, there may be many complex reasons why the weather is so cold - but all these scenarios can be improved by putting on warm clothes. A suitable reward function could be the difference from optimal body temperature, which is easily measured. It is then easy to learn that reward increases if you put on a sweater when you are cold.

### The Memory-Prediction Framework

The Memory-Prediction Framework (MPF) is a general description of a class of pattern recognition and classification algorithms. MPF describes an unsupervised learning system - there is no objective except accurate modelling. MPF was developed by Hawkins and Blakeslee [5] as an attempt to describe the function of the human neocortex. A successful implementation, known as Hierarchical-Temporal Memory (HTM), was first produced by George and Hawkins [6]. An open-source implementation of MPF/HTM has been produced by Saulius Garalevicius [7]. Both MPF and HTM are auto-associative memory systems consisting of tree-like hierarchies of pattern-classifiers. Within each unit of the hierarchy, data is compressed by the discovery of spatial and temporal patterns (spatial and temporal “pooling”). Messages about these patterns are transmitted between levels in the hierarchy.

HTM extends MPF by borrowing belief-propagation techniques from Bayesian Networks [8], [9]; the data transferred between nodes are likelihood or probability mass functions over a set of states defined within each node [10] [11]. The literature includes many similar cortical simulations using Bayesian Belief-Propagation in hierarchical networks, such as [12] and [13].

**Figure 1:**
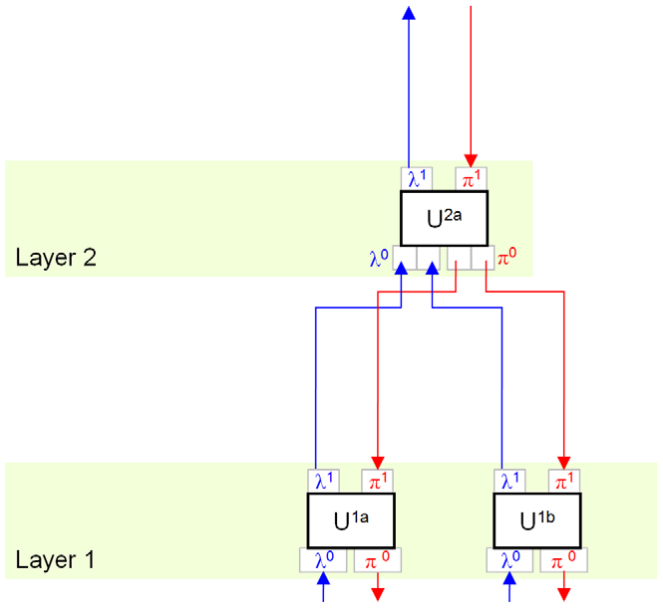
Hierarchic structure of the memory-prediction framework. Blue (upward) arrows show the flow of data from lower layers to higher layers in a feed-forward (FF) traversal. Data from multiple children may be concatenated, giving the hierarchy a tree structure. Data in higher layers has a greater number of invariances. Red (downward) arrows show the flow of data in the feed-back (FB) traversal. The FF pass performs classification; the FB pass generates predictions. Each unit has two inputs and two outputs. 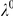 is the FF input; 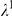 is FF output. 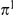 is FB input and 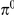 is FB output.

It is important to understand some properties of the MPF/HTM hierarchy (figure 1). First, raw data is presented at the “lowest” level. Classifications are extracted at the “higher” level(s). The hierarchy is traversed in two directions, feed-forward (FF) and feed-back (FB). In the feed-forward pass, raw data from the lowest level is incrementally quantized and compressed at each level, until it reaches the highest level in the hierarchy. Active “labels” in the highest level of the hierarchy represent classifications of the raw input.

In the feed-back pass, activity in the highest levels is transformed and expanded at each level until it becomes a pattern of raw data at the lowest level. The MPF/HTM hierarchy is expected to find patterns in space (between inputs, via spatial pooling) and time (coincidences and sequences, via temporal pooling). Due to pooling, data at higher levels in the hierarchy are increasingly invariant over time and space. The accumulation of invariances is analogous to the production of increasingly symbolic representations.

Temporal pooling allows the hierarchy to generate predictions. This occurs because temporal pooling causes states in higher units to represent sequences observed in lower units. It means that higher units can't represent the state of lower units accurately, but with the benefit that classification in higher units translates into sequences of states in lower units. These sequences can be used as predictions.

For example, if a lower unit regularly observes state sequence A,B, a higher unit will form a model X that represents A,B. When the higher unit recognizes the current state as X, this message is translated into states A and B when passed to the lower unit. Assuming the lower unit is already in state A, the message allows the lower unit to “expect” state B. In HTM the feedback data represent a belief of the state of the unit around the current time, given all the data available higher in the hierarchy, including observations from sibling units. In this paper, addition of a predictor within each unit further biases feedback data towards future states of the unit. At the lowest levels, data in the FB pass become a prediction of FF input in the near future. In this way the hierarchy is capable of both classification and prediction.

More recently, Hawkins, George et al have developed a very detailed understanding of how HTM could be implemented in biological neural networks [11] and there have been a number of successes using HTM for optical character recognition [14], [15], image classification [6], [11] and spoken digit recognition [16]. Other impressive HTM demonstrations include the classification of observed human motion into categories such as walking and sitting [17], preliminary studies on human motion classification and reproduction [18], and music classification and production [19]. These tasks are generally considered difficult for machines.

A greater variety of literature is relevant if HTM-like methods are included: For example, Morse [20] [21] described ESNs, recurrent neural networks that create hierarchical models similar to HTM. They used SOMs (see below) to compress data between levels, and explored the relationships between actions and perceptual learning. But there has been no attempt to use MPF or related methods in an adaptive agent.

### Exploiting the Hierarchy

A key difficulty in creating an adaptive intelligent agent is that easily measured internal physical conditions such as pain can have many causes and require complex sequences of actions to fix. Often, it is necessary to identify and understand quite abstract concepts - such as the identities of people with different personalities - to successfully predict what will be nice or not. Similarly, some threats or opportunities may not be continually observable, or may take a long time to develop. The ability of the MPF/HTM hierarchy to construct increasingly time-invariant and abstract representations suggests that it would be an effective perception and prediction system for an adaptive intelligent agent, whose internal objectives are affected by many complex relationships with its world.

Since knowledge and understanding of agent-world interactions in a MPF/HTM model would be distributed throughout the hierarchy, it is not immediately obvious how MPF/HTM can be included in an adaptive system. Before learning, it is not possible to predict what objectively-useful concepts will exist, or where they will be found in the hierarchy. It would be possible to attach an entirely separate adaptive control system to specific levels in the hierarchy, but then behaviour would be generated outside the hierarchy rather than within it. Since this separate system would have to duplicate a lot of the knowledge already in the hierarchy, it would be inefficient. It is also biologically realistic to expect complex behaviours to be generated within the MPF/HTM hierarchy, if it is an accurate analogy of the human neocortex.

To generate adaptive behaviour within an MPF/HTM hierarchy, it is necessary to use information at all levels because (a) details of the current state are distributed between many levels, and (b), as activity moves from higher to lower levels, behaviours are refined and given increasing detail both spatially and temporally. For example, an abstract behaviour such as “drink” must be translated into a series of coordinated movements to successfully end discomfort caused by “thirst”.

With these points in mind, our objectives are:

- To allow the MPF to perform as an adaptive control system while simultaneously continuing to perform hierarchical learning and prediction
- To introduce minimal changes, e.g. not introducing a model to translate the state of the MPF into another form
- To not restrict the organisation of the MPF hierarchy
- To generate and model agent behaviour within the MPF hierarchy, not in a parallel model
- To effectively exploit information anywhere in the hierarchy, without prior knowledge of where concepts will exist in the hierarchy, or what they will represent
- To rely on the MPF discovering and modelling relationships between complex causes, simplifying the adaptive component of the system

### Hierarchical Reinforcement Learning

In the literature, approaches to hierarchical reinforcement learning share the same basic approach of defining macro-operators that represent sequences of simpler actions [4], [22]. This fits neatly into MPF, where increasing temporal pooling will naturally form states representing sequences of actions from lower layers.

Skelly [23] describes some of the expected benefits of hierarchical reinforcement learning: “… using greater abstraction will be shown to require less experience from state transitions and rewards in the environment, because the generalization helps make maximum use of each experience by diffusing the information from each experience to the local region of the state and action space where that information is meaningful”. Barto and Mahadevan offer another advantage, that hierarchies “combat dimensionality by exploiting temporal abstraction where decisions are not required at each step, but rather involve execution of temporally extended activities that follow their own policies until termination. This leads naturally to hierarchical control architectures and learning algorithms” [22].

The majority of Reinforcement Learning (RL) problems can be collectively best understood as the search for optimal solutions to Markov Decision Processes (MDPs) [4]. A solution is defined as a “policy” for choosing actions that maximises cumulative future reward. In the hierarchical case, the Semi-MDP (SMDP) formalism is adopted [22], [24]. SMDP models the time interval between decisions as a random variable, describing the durations of macro-actions composed of shorter actions.

Most hierarchical approaches to RL require the hierarchy of possible actions to be defined in advance [22], [25]. In this paper we allow the MPF to define a hierarchy of state-action pairs, and allow transition probabilities to be adjusted by reinforcement learning.

### Planning as Inference

A stationary Markov Decision Process (MDP) is one where states, state transition probabilities and rewards for specific states do not change over time. Most RL algorithms are only suitable for finite state/action spaces and stationary MDPs in which the set of possible actions and consequent rewards are fixed.

A Partially-Observable MDP (POMDP) [4], [26] is an MDP in which the state of the world cannot be known accurately. Typically, approaches to POMDPs involve assigning probabilities to observations and updating a probability distribution over an underlying set of states.

The stationary MDP criterion means that for most hierarchical RL algorithms, including HAMs,MAXQ and ALisp, the set of macro and atomic actions must be defined a priori [22], [25]. We propose instead that unsupervised learning within MPF can define a MDP of actions and macros, but it will evolve over time due to unsupervised learning and adaptive bias.

In this case, the problem becomes a POMDP. In general, finding globally optimal mappings from observations to actions in POMDPs is NP-hard [27] but some locally-optimal solutions have been proposed [28]. McGovern and Barto [29] have investigated the problem of constructing hierarchical representations of actions based on the frequency of successful “trajectories” (sequences of tasks). This approach is similar to our correlating method.

More recently, several authors have tried to reformulate planning by reinforcement learning as an inference problem. This allows use of a variety of well known inference methods such as Expectation-Maximization (EM) and Markov-Chain Monte-Carlo (MCMC) [30]. Attias [31] describes an algorithm with separate modes for exploration and “exploitation”, the latter meaning using learned models for goal-directed navigation. Exploration is necessary to build models of possible action sequences, and is implemented by sampling from a fixed prior distribution over possible actions. In exploitation mode, planning consists of finding an action sequence that maximizes the posterior distribution conditioned on arrival at a goal with a fixed number of steps. Both MDP and POMDP results are presented. In the POMDP case, the posterior is conditioned on initial observations and the final (goal) state.

Toussaint and Storkey [32] define solving an MDP as likelihood maximization in a variable mixture model. This permits use of Expectation-Maximization (EM) to search for mixture models that maximize discounted expected future reward over an infinite horizon, avoiding one of the most significant limitations in Attias' work. They demonstrate both discrete and continuous action-space MDPs.

Subsequently, Vlassis and Toussaint [30] extended this approach to POMDPs using an approach called Stochastic Approximation EM (SAEM), but this only guarantees convergence to local optima and only in certain conditions. Their methods are very relevant to solving the POMDP defined by the states and action-sequences within the hierarchical MPF.

### Structure of this Paper

In this paper we describe and demonstrate a way to generate adaptive behaviour within an MPF hierarchy by modifying (biasing) inter-unit messages during the feed-back pass through the hierarchy. The changes cause the MPF to preferentially predict states where its output causes actions associated with higher reward from a hidden objective function. Crucially, we allow associations between hierarchy states and internal reward to be generated at any or all levels in the hierarchy, wherever a strong correlation can be found. The remainder of this paper is presented as Methods, Results and a Discussion.

In “Methods”, we first describe how both sensing and actuation can be connected to the MPF hierarchy. Next, we describe additional components required to make a hierarchy adaptive. We develop the concept of the MPF as part of a reinforcement learning system, with the impact of external causes being felt through a single reward function and understood by hierarchical modelling.

“Methods” also gives a detailed description of our implementation of an MPF unit. The unit performs both spatial and temporal pooling, and internal prediction (sequence learning). We discuss implementations of both first-order and variable-order sequence learning. In “Results” we present several demonstrations of the ideas within the paper.

## Methods

### Sensor-Motor Interface

**Figure 2:**
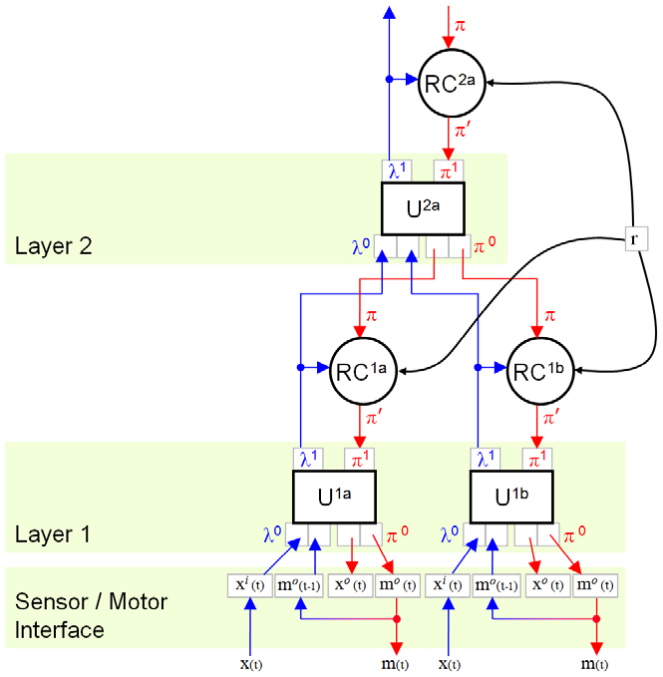
Formulation of an adaptive-MPF hierarchy. Messages between units (U) in different layers are relayed via “reward correlator” components (RC). FF messages (blue arrows) represent classifications of the current state of the agent in the world; these are correlated with objective internal measures of agent state (reward). The same reward value 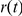 is provided to every RC; the hierarchy is tasked with modelling the separate external causes of changes in reward. FB messages 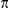 are “predictions” of future agent-world state (red arrows). Biased messages 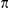 are produced by RC components, making the hierarchy more likely to “predict” states in which it performs actions correlated with high reward. Sensor data 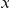 is concatenated with motor output 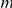 to form the interface to the MPF hierarchy. The FB output of an MPF unit is of the same form as its FF input. Different data may be presented to each unit at the bottom of the hierarchy. Sensor inputs and motor outputs may be mixed within one unit or interfaced to different units.

We operate our hierarchy iteratively. Each iteration includes a feed-forward (FF) pass of every unit, followed by a feed-back (FB) pass of every unit. The units are traversed in such an order that all units at the lowest level are FF prior to any unit at the next higher level (breadth-first or level-order traversal). On the FB pass, the units at the highest level are FB prior to any “lower” units, until the lowest level is reached. Each iteration therefore consists of a FF and a FB pass of the entire hierarchy (figure 2). Although synchronous operation of the hierarchy is not biologically realistic, it should not affect the results of the algorithm described below.

Each unit has 4 data structures for input and output. Let 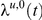 be the FF input to unit 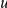 at iteration 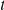, a vector of real numbers in the interval 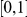. In higher layers 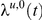 is a mass function, but in the lowest layer any values in this range can be provided. Let 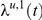 be the FF output, a matrix containing a normalized likelihood function over possible classifications of the input 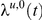 within 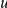. For the FB pass, let 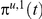 be a matrix of equal dimension to 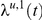 containing a probability mass function over predicted future classification-states in unit 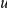. Similarly, let 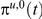 be the FB output from 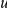, a vector of equal size to the input vector 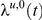 containing a prediction of future input to unit 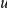. To form a hierarchy, FF outputs from multiple lower units 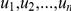 are concatenated and presented to higher unit[s]. Conversion from matrix to vector is not important as the classifiers (see below) assume all input dimensions are independent.

### State-Action Pairing

Many reinforcement learning algorithms - such as Q-learning [3] and SARSA [33] - model the effect of [state,action] pairs on reward. The state contains both external and internal measurements from the agent in its world. Actions are generated by the agent. The expected reward of performing actions 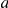 when in state 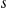 is the “Quality” of the pair, typically denoted 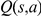.

Since in MPF the FF and FB data structures are of equal size, agent sensor values and motor commands must be present in both 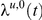 and 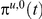. If 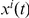 is a vector of current values from the agent's sensors, and 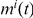 is a vector of values corresponding to motor commands, then 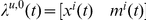. In other words, the input/output state to the MPF's lowest level is a concatenation of sensor values and motor commands.

However, in an iterative artificial adaptive agent 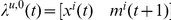, meaning that the state is comprised of current sensor values and consequent actions taken. The agent must learn which action to choose given that it is in a particular world/self state, so we must store this combination together. Imagine the Markov-Graph of this model; we are encoding the current vertex and outbound edges, rather than the current vertex and inbound edges (learning how we got into a nasty situation is not as directly useful as learning how to get out of it!). This is similar to the state-action pairing seen in SARSA.

We want the MPF to generate behaviour directly. If 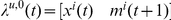 then 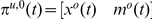. Given the behaviour of the MPF, 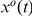 will be a prediction of 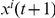 and 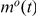 will be a prediction/suggestion of motor commands at 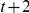; i.e. when trained, 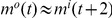.

If continuous motor outputs are desired, values in 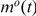 can be used without further processing. Discrete outputs are more problematic because learning within the MPF unit (within a SOM in this paper) will cause a feedback loop, pulling motor outputs towards intermediate values. Instead, discrete outputs can be produced by sampling from a multinomial of possible actions with probabilities 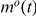. 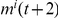 should represent the action actually chosen from 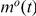. Therefore let 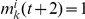 if action k was chosen, and 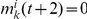 otherwise.

The different problems of discrete and continuous outputs (action spaces) are discussed in the Reinforcement Learning literature. Many RL algorithms (such as Q-learning and SARSA) cannot handle continuous action spaces. However, approximately optimal continuous outputs can be learnt by methods such as CACLA (Continuous Actor-Critic Learning Automaton) [26]. The RL literature does include Monte-Carlo methods to explore the space of possible actions (policies) [28], similar to our approach for discrete outputs.

### Additional Adaptive Components

#### Reward Function

The adaptive-MPF system uses reinforcement learning rather than supervised learning because we do not wish to provide “correct” outputs for every conceivable situation. Instead, we wish to measure impacts of external causes on properties of the agent, such as pain or hunger. In this paper we will use the simplest possible reward function, providing a single varying scalar value 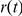 such that 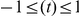. We use the term “reward” to imply that this function should be maximized. We will expect the MPF to build a model of the world that is capable of understanding the causes of changes in reward. By combining all possible definitions of things good and bad within a single scalar, it becomes much harder for the MPF to learn the separate causes of high and low reward.

Since the agent should be highly motivated to improve a bad situation, it is more useful to maximize the first derivative of reward. We also wish to measure changes in reward over a period of time, because the delays between actions and their consequences are varying and unknown. However, it is more likely that recent actions are responsible for changes in reward. Over a few iterations, this can be approximated simply as an exponentially-weighted moving average:
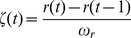
(1)

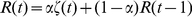
(2)

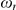
 is the maximum possible absolute derivative of reward per iteration, if known. The 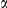 parameter is determines the influence of historic reward signals. If consequences of actions may take some time to be reflected in 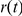, the value of 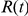 should instead be computed over a window of time. The period should be increased for units at higher levels in the hierarchy, whose state changes very slowly.

This differs significantly from conventional reinforcement learning where a “discount” factor allows future reward signals to be considered when evaluating state-action pairs [4]. Rewards further in the future have less influence, and are therefore said to be “discounted”. Many RL algorithms iteratively propagate discounted rewards backwards in time towards the events that caused them.

In this paper we rely on the existence of an arbitrarily deep hierarchy with increasing temporal pooling, to avoid the need to consider discounted future rewards. We assume that for any event with delayed reward there will exist a level in the hierarchy that remains constant for the duration of the event-reward interval. For example, a state in the hierarchy corresponding to a high-level plan such as “walk the dog” could be active for long enough for all relevant rewards to be integrated, despite the existence of other transient plans during this period. This is unlikely to be an ideal approach and in future work we will investigate the use of discounted future reward.

#### Reward Correlation

Since we have defined that data inside the MPF hierarchy includes representations both of [sensed world-state] and [agent motor-actions], it should be possible to correlate activity patterns within the hierarchy with the reward values that result from the agent taking specific actions in specific situations. While it is necessary that concepts with appropriate abstractions and invariances exist somewhere in the hierarchy, it is not desirable to have to define where, before learning. We also wish to preserve the homogeneity of the MPF, therefore it must be possible to add the adaptive components throughout the hierarchy without negative effects.

**Figure 3:**
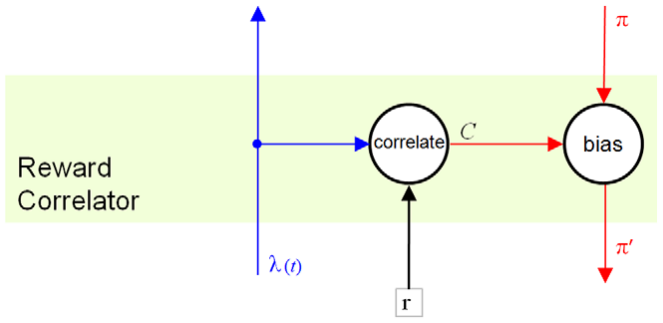
Reward Correlator component. In the FF pass, delayed lower unit output 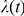 is correlated with reward 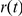. The FF message is then relayed, unaltered. Correlations are stored in matrix 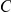. In the FB pass, higher unit messages 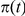 are modified to bias them towards states correlated with high reward. The modified message 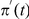 is then relayed to lower units.

In this paper we suggest that correlation of activity patterns with reward values could occur between layers of the hierarchy. We posit a “reward correlator” component that relays messages between units in different layers, i.e. matrices 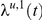 and 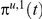 are inputs and outputs of a reward correlator above 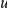 in FF and FB passes respectively (figure 3).

The FF pass through the hierarchy should classify the current state as accurately as possible. The purpose of the FB pass is to generate predictions, and as a result, behaviour. We choose to modify messages between units in the FB pass, causing the MPF to preferentially “predict” states where its output causes actions associated with higher reward. More specifically, in the FF pass we correlate matrix 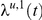 with scalar 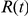 and in the FB pass we modify matrix 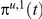. Since there is a feedback loop within each MPF unit (detailed below), an alternative arrangement would be to correlate 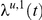 with 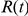 and modify 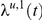 prior to relaying it to higher unit[s].

For every unit 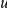, if 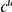 is a correlation 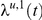 matrix of equal dimension to 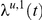 and 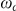 is a scalar learning-rate parameter (gradually decreased over time), then we define a temporary matrix 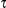 to correlate:
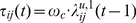
(3)

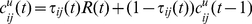
(4)


This formulation arises because we only want to change the correlation for active elements in 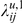 and the influence of 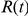 on any element 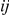 should depend on the probability that SOM model 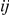 represents the state that caused 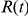. 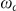 ensures that the correlation never changes too quickly, forgetting historic values. If events happen comparatively quickly compared to the rate of iterating the hierarchy, a delay of at least 1 iteration should be applied to the correlating formula as shown above, although 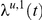 should be relayed without delay to higher units. In more sophisticated implementations, integrals of 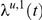 over time should be correlated with reward.

#### Adaptive Bias

In the FB pass we wish to modify message 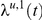 passed from higher unit[s] to unit 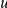. Since 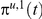 is a probability mass function, we wish to increase the value (mass) of matrix elements associated with increase in reward, and reduce elements associated with decreases in reward. This can be done with the following formulas, in which 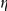 is a normalizing factor ensuring constant mass and 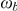 is a global scalar parameter determining the maximum influence of adaptive bias 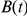. Note that matrix 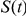 is a nonlinear function of the correlation of unit states with reward, to ensure that weak correlations are rapidly tested and either strengthened or depleted. 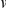 is a scalar constant corresponding to the uniform mass value i.e. 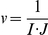 if 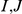 are the dimensions of 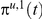. 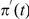 is the modified mass function:
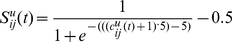
(5)

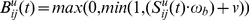
(6)

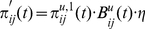
(7)


### SOM-MPF Implementation

Thus far we have described several additions to the Memory-Prediction Framework to enable it to be used as a complete control system for an adaptive intelligent agent. In summary, we have reformulated the input/output data to include sensors and actuators, and are modifying FB messages between hierarchy layers, by correlating delayed FF messages with smoothed reward signals (figure 2).

#### MPF Unit Structure

**Figure 4:**
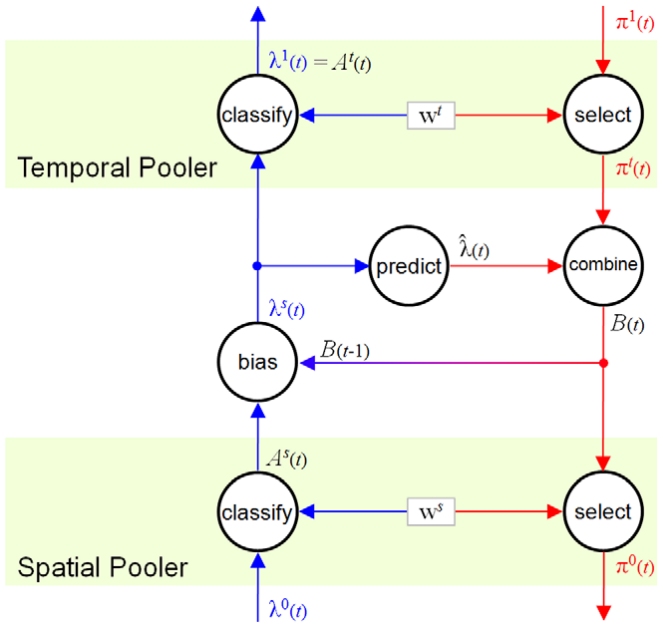
Internal structure of a SOM-MPF unit. In the FF pass, SOM and RSOM components perform spatial and temporal pooling (compression) by classification of 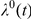 in terms of the finite sets of models 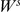 and 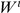. FF SOM classification output 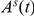 is biased by previous prediction 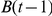 resulting in 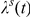. 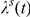 is the FF input to the RSOM temporal pooler. RSOM FF output 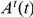 is used as unit FF output 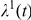. Between the poolers, an internal loop estimates unit state in terms of SOM models, using both 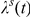 and FB predictions from higher units via the RSOM. Internal predictor output 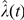 is combined with RSOM FB output 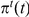 to give the bias matrix 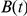. Predictions from higher units indicate the current sequence, as in HTM; predictions within the unit allow position within sequences to be tracked also. In the FB pass, roulette selections from 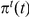 and the combined PMF 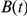 are used to reverse the RSOM and SOM transformations, giving unit FB output 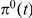.

Although the above modifications should be compatible with various implementations of MPF (including HTM), we will describe our implementation of the MPF unit. Each unit performs spatial pooling, sequence learning plus prediction, and temporal pooling. We will discuss each of these components (figure 4).

Our MPF unit is based on the Kohonen Self-Organising Map (SOM) [34], and unlike some HTM solutions, is capable of online learning. The SOM is a biologically-inspired artificial neural network used for unsupervised classification and dimensionality reduction. Others have previously used SOMs to build MPF-like hierarchies, such as Miller and Lommel's Hierarchical Quilted SOM (HQSOM) [35]. Pinto [36] extended this to a complete MPF implementation. Other SOM variants could equally be used.

The chief innovation in Miller and Lommel [35] is use of a “Recurrent”-SOM (RSOM) that can perform temporal pooling (clustering) by allowing current classification to be affected by previous classifications. Therefore, a SOM-RSOM pair can perform both spatial (SOM) and temporal (RSOM) pooling, as described in the MPF.

#### Feed-Forward Pass: Spatial and Temporal Pooling

The SOM consists of two matrices 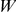 and 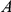. 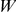 is an 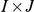 matrix of models of the input vector 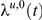 such that given 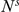 elements in 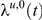, the dimensions of 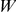 are 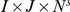. 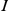 and 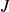 are parameters that determine the number of models the SOM will contain. In this case the SOM has a 2-d topology, which is usually sufficient but cannot optimally represent all data. 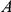 has size 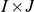 and 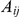 represents the likelihood of observing the SOM model 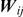 given the evidence 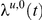. Each SOM model represents a possible configuration of 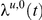 and the models in the SOM learn to maximize their coverage of the input space observed in 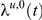 over time. Since the SOM has been thoroughly discussed in many works, the reader should consult e.g. [35] or [36] for detailed SOM weight update equations. For our purposes we define the likelihood function as the inverse of normalized sum of squared error, giving matrices 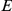 and 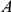:
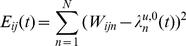
(8)

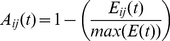
(9)


These equations produce a very smooth result, with significant responses from many models within the SOM. This is desirable because we wish to bias the FF classification result using a matrix 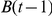 that was produced in the previous FB pass. 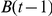 is a probability mass function representing a (biased) prediction of 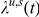, the spatial pooler classification:
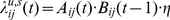
(10)

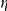
 is a normalizing constant such that 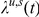 becomes a probability mass function over the classification-states represented by the spatial pooling SOM models. The superscript ‘s’ indicates that this is the FF output of the spatial pooler in unit 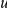.

According to MPF, the FF output of the spatial pooler (SOM) should be the FF input to the temporal pooler (RSOM). Since the RSOM and SOM treat all input dimensions independently, we can rearrange the SOM output matrix to become a vector of 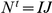 elements. However, as discussed in [35], the RSOM input should be highly orthogonal. This can be achieved by setting the maximum value in 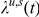 to 1 and others to zero. For other details of the RSOM, see [35]. The FF output of the unit would typically be the FF output of the temporal pooling RSOM:
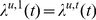
(11)but in hierarchy layers where a lot of spatial compression is required (e.g. in the visual cortex) the temporal pooler can be omitted. In this case the unit FF output is taken from the spatial pooling SOM:
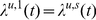
(12)


In [37] it is noted that in higher layers of the hierarchy, there is little or no advantage to further spatial pooling. This is believed to be represented in biology by the absence of neocortical layer 4 [37]. To reproduce this effect, in these units the spatial pooling SOM can be omitted.

The classification process in the RSOM is similar to the SOM but functions as a “leaky-integrator” so that classification outcome changes slowly. A matrix 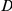 of equal dimension to 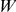 is needed:
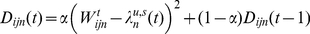
(13)

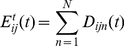
(14)


It is not necessary to bias the RSOM classification result, either using a prediction or for adaptive purposes. This asymmetry is because in the FF pass, active RSOM sequences become spatial patterns in a higher unit, where they can be predicted. In the FB pass, adaptive selection between RSOM sequences translates into preference for sequences containing better spatial patterns. Hence:
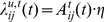
(15)

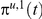
 is therefore a normalized likelihood function if an RSOM is used, or a probability mass function otherwise.

#### Prediction and Sequence Learning

The SOM-MPF units used in our experiments include either first-order or variable-order Markov prediction. The MPF framework does not require a prediction feature, as temporal pooling generates predictions of proximate future and past states in the FB pass. However, our prediction module predicts only future states, which reduces uncertainty within the system. It also allows units to track position within sequences. For prediction and sequence learning, an MPF unit should do three things: identify the set of observed temporal sequences, classify the current temporal sequence, and predict future sequences.

Both first-order and variable order variants of HTM have been developed. The benefit of variable-order prediction can easily be illustrated: A 2nd (or higher) -order model can distinguish between 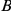 in sequences 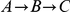 and 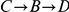, whereas a 1st order model cannot. In [17], Hawkins et al use a Variable-order Markov Model (VMM) to implement the temporal pooling stage of HTM. However, they note that even with a VMM, the hierarchy must be used to distinguish between longer intersecting sequences. (The hierarchy allows assembly of longer sequences from shorter ones). The difference is flexibility and efficiency; a VMM-hierarchy can distinguish longer sequences using fewer layers.

In our SOM-MPF implementation we present a first-order Markov Model to predict future classification outcomes. Later, we also show how using the biologically-inspired technique described in [17], we can adjust the first-order model to behave as if it were a variable-order model.

#### First-Order Prediction

The input to the prediction module is 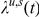 and the output is a matrix 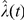 of the same size. Both are probability mass functions. 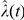 is a prediction of 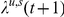. 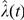 is generated from a matrix 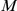 of size 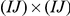 (i.e. each model in the SOM is treated independently and regardless of SOM topology). 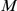 is updated using 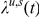 and 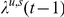 and approximates the conditional probability of SOM model 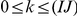 being active at 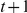 given that model 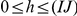 is active at time 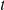. The sum of each column of 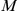 is normalized to 1.
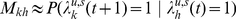
(16)

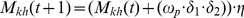
(17)where 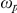 is the learning rate (typically 0.99 initially and reduced to around 0.01 over time) and:
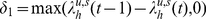
(18)

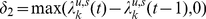
(19)


Equations 17, 18 and 19 increment the conditional probabilities in 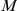 if 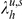 is observed to decrease while 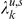 is increasing (a transition between 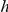 and 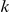). Since 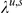 is a probability mass function, a reduction in mass at 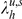 is interpreted as the exiting of state 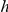. Similarly, an increase in mass at 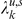 represents entering state 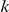. These equations are best understood as approximating transition probabilities by computing the relative frequency of transitions between states. The relative frequency of an event becomes closer to the probability of an event as the number of trials increases. However, in this case the approximation is biased towards recent events by 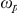. Since the underlying system is continually changing (due to SOM learning), frequency-based approximation biased towards recent data is simple and effective.

A first-order prediction can be obtained from 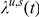 and 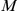 by:
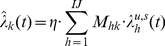
(20)


Matrices 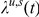 and 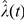 are treated as vectors in equation 20. 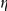 is a normalizing constant giving a total mass of 1:
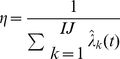
(21)


#### Feedback Pass

In MPF, the purpose of the FB pass is to generate a prediction of the next FF pass. MPF proposes that FF classification be combined with FB prediction, yielding more accurate models of the world given noisy, indeterminate or insufficient data. Feedback also improves state estimation between sibling units, via higher units. In this paper, the purpose of the FB pass is also to generate adaptive behaviour.

The FB pass through the unit starts with 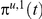, a prediction from higher units in the hierarchy. (For the highest layer 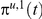 can be a uniform distribution). 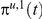 is a probability mass function over the set of models in the RSOM. “Roulette” selection is used to select an element 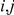 from 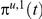 (i.e. the probability of the selection of any element 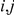 is proportional to the value of 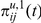). The corresponding weights from this RSOM model are then copied to a matrix 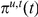.

The use of Roulette selection for the selection of a FB model from the (R)SOMs is unique to this paper and has some useful properties. If there are multiple modes in 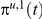 the FB pass will test them individually, until one fits. When a mode in 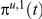 accurately predicts reality, it will rapidly be reinforced by the feedback loops within the unit and hierarchy, and the mass of the other modes will decrease. More importantly, there is no guarantee that interpolating between the models in the SOM generates viable patterns, therefore a weighted-sum of the models in is 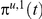 not effective given high variance modes or a multimodal case (in practice, multi-modal distributions are quite common). Using clustering techniques to find a single mode in 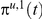 is more expensive and in our experiments gave no noticeable improvement. Over time, the series of selections from 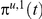 can be interpreted as a probability mass function because the normalized likelihood functions represented by the SOM models are conditioned on the distributions in 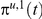. The kurtosis of the distribution in 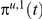 balances the conflicting demands of exploration and exploitation; if the distribution is flat chosen actions will be more random (i.e. exploratory).

We wish to generate all behaviour within the MPF hierarchy and not require any external module to help control the agent. However, the MPF must explore the gamut of possible action-sequences, motor outputs etc. and learn their consequences. This objective is achieved both by using Roulette selection of individual SOM models, and by adding random noise to the models in the FB pass. Let 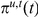 represent the roulette-selected model from the RSOM. To add noise:
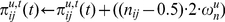
(22)


The magnitude of the noise is scaled by 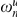, a parameter that should initially be 1 and decreased over time to a low value (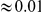). All results are clamped to unit range. The schedule for reducing noise magnitude should consider the location of the unit within the hierarchy; higher units inputs' are not well defined until lower units have learned. 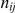 is a uniformly distributed random value from the interval 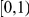, as produced by most software random number generators.


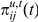
 is a mass function of the same random variables as 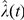. They are both predictions of the outcome of the next FF classification from the SOM. We combine them using the element-wise product and add a small uniform mass 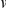 to every element, giving us the bias matrix 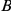:
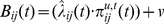
(23)

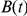
 will be used in iteration 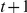. The uniform mass serves to introduce some plasticity and uncertainty into the system even when it has modelled predictable data very accurately. It also prevents numerical instability when predictions from higher layers do not agree with predictions within the unit, or when the final bias does not agree with observed reality. There is a fundamental conflict between the objectives of accurate prediction and adaptive bias; by definition, adaptive bias disrupts - damages - the prediction process. It is also important that biased classification in the FF pass does not become locked into an internal loop, ignoring observations from below.

The final step in the FB pass is to transform 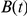 into so that the message can be passed down the hierarchy. This is achieved by using “Roulette” selection to pick a SOM model 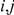 from 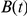 and adding noise:
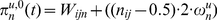
(24)


#### Variable-Order Prediction

To increase the representational flexibility of the hierarchy, we will describe a modification to the first-order prediction component that exploits the 2-d topology of the SOM. The technique is inspired by Hawkins et al's article on Sequence Memory [17]. They describe two levels of organisation for cells in the neocortex: A “region” is a group of cells receiving the same input. “Clusters” or “columns” are groups of cells within a region, that respond to the same input patterns.

**Figure 5:**
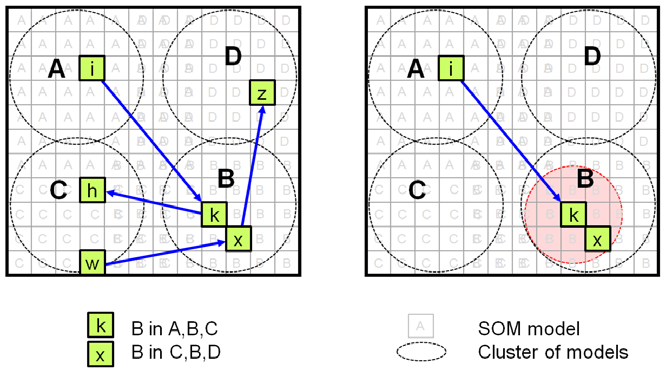
Variable-Order prediction. This figure shows two views of the models within a SOM. Each small square represents one SOM model. Clusters of models (indicated by dashed-circles) respond to the same input pattern, in this case the letters A,B,C or D. The clusters are implicit; they are a consequence of models' content. If several models respond to each letter we can individually specialize them to respond to occurrences of the letter in specific sequences. This is achieved by developing strong first-order edges between individual models (blue arrows). For example, a high weight on the edge 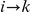 represents 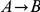. Assignment of SOM models to specific occurrences of letters occurs by inhibiting unpredicted neighbours of predicted models. In the right panel, activation of SOM model 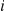 at time 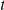 promotes 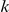 via the first-order edge 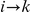 and inhibits 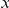 and other nearby models at 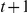. The neighbourhood of inhibition is indicated by the red circle.

They suggest that within each column of cells there is a “winner” that locally-inhibits other cells. The winning cell is (typically) pre-activated by connections from other columns. These connections allow the encoding of sequences. Although each cell's response is not unique in terms of input pattern, it is unique in both pattern and sequence. Hawkins' example uses letters; having several cells that respond to the letter ‘B’, one might respond to ‘B’ in ‘ABC’ and another to ‘B’ in ‘CBD’. This can be most clearly explained in a diagram (see figure 5).

These groups of “cells” are analogous to clusters of similar models in the SOM weights matrix 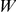. The topological constraints in the SOM weight update equation ensure that models responding to similar input patterns are located together. Within each cluster, we can use local inhibition to ensure that only one SOM model responds. We can simulate the connections from other columns by inhibiting models with strong first-order relationships, whose priors were not activated.

These modifications can all be made within the prediction module, because prediction affects FF classification outcome via 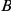. (This is the same biasing process that allows the SOM-MPF hierarchy to be adaptive). In addition to modifying predictions to become adaptive, they will be modified to encourage the formation of variable-order sequences within each unit. This reduces predictive accuracy in the short-term, but once variable length sequences are learnt it can lead to superior predictive ability within a single unit. This result is shown in one of our experiments, described later.

Variable-order prediction is implemented by adding preprocessing and postprocessing steps to the first-order prediction system described above. Preprocessing consists of a local inhibition around the global maximum in the input matrix 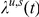, implemented using a Difference of Gaussians (DoG) function centred on the maximum value. This ensures a clear winner within the “column”:
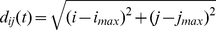
(25)

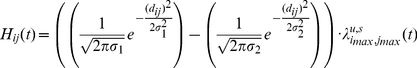
(26)Values 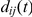 are temporary scalar quantities to improve readability. 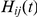 is a matrix of size 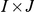. The values of 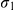 and 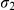 are related to the SOM's weight-update 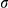. If online SOM learning is used,
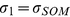
(27)

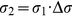
(28)where 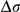 is a parameter greater than 1 such that in all cases:
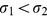
(29)


The values in 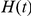 are linearly scaled to occupy the range 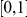, and then the element-wise product gives the inhibited predictor input:
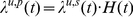
(30)

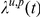
 must be renormalized to have a total mass of 1.

Postprocessing involves two steps. First, elements for which a strong first-order prediction exists are inhibited if that prior element was not active in the previous iteration. Second, around inhibited elements, neighbouring elements are promoted. This is because SOM topology ensures that these will respond to similar input patterns, and are viable alternatives.

Intuitively, the inhibition is a nonlinear (log-Sigmoid) function of the product of first-order weights 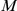 and preprocessed prediction input; it is maximized when the prior values for high-probability Markov Graph edges are small (“if A implies B and A is not observed, inhibit B”). Again we treat the matrices as vectors to simplify indexing, and compute 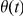, a matrix of equal dimension to 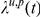.
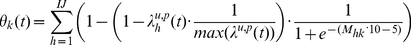
(31)


The values in 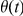 are linearly scaled to occupy the range 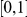, then using the element-wise product:
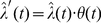
(32)


We compute a matrix 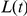 of equal dimension to 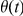 containing the mass lost by inhibition:
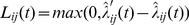
(33)

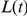
 is used to construct a “local-promotion” matrix 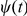 which increases the activation of neighbours of inhibited elements. The promotion matrix is computed using a Difference of Gaussians (DoG) function:
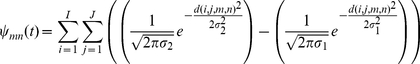
(34)where 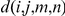 is the Euclidean distance between coordinates 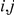 and 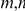. 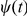 is linearly scaled such that:
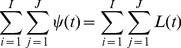
(35)and then added to 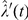:
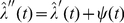
(36)


These modifications cause the modified prediction to interfere with the FF classification process, forcing individual SOM models respond to patterns only at specific points in variable length sequences.

## Results

To demonstrate the methods described in this paper, it is accompanied by open-source code and a compiled program that can run 4 separate demos. The code and program can be downloaded from: http://code.google.com/p/adaptive-memory-prediction-framework.

The code is provided as a toolkit, enabling interested readers to develop their own tests. The software is written in Java, and requires the JDK or JRE to be installed. To run the demos, download the .jar file and execute with the following command:
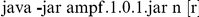
where 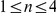 is the number of the demo and ‘r’ is optional. If ‘r’ is omitted, the random number generator uses a fixed seed. The first demo exhibits the SOM and first-order predictor components. The second demo is a reproduction of the moving-line recognition program from Miller et al [35], which uses a SOM-RSOM pair. Third, we show that a SOM-MPF unit comprising SOM, RSOM and variable-order prediction can distinguish words such as ‘dad’ and ‘bab’ - the classic variable-order problem described in Hawkins et al [17]. The fourth demo uses a very simple hierarchy of 2 SOM-MPF units connected via a reward-correlator. This hierarchy can be seen to successfully play “rocks, paper, scissors” against a predictable software opponent.

### Demo 1: RGB-SOM-1MM

**Figure 6:**
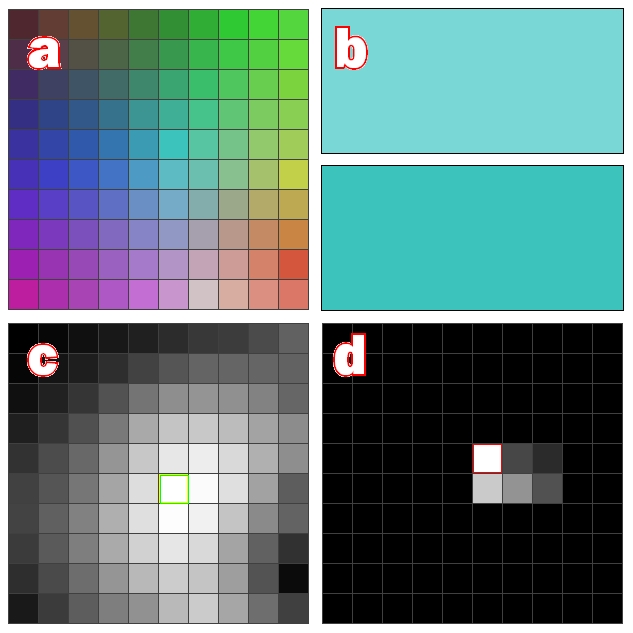
Screenshot, Demo 1. (a) SOM weights matrix 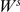; each of the 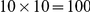 SOM models has r,g,b values. (b) Upper box shows input colour. Lower box shows colour predicted during previous FB pass, so it should be similar to the colour of the upper box. (c) SOM activation 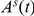 (shown scaled to full greyscale range). Matrices are displayed such that brightness indicates mass or value (i.e. white = 1). The best-matching SOM model is outlined in green. (d) 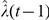, the output of the predictor in the previous pass. Since the external RGB process is stochastic and classification quantizes FF input, prediction is not precise.

The first demo provides an intuitive visualisation of the SOM. The input vector 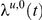 is a 3-tuple in RGB space (i.e. 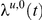 = 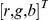), and the SOM constructs a 2-dimensional representation of this 3-dimensional space (visible in figure 6, panel (a)). Each successive input colour is classified by the SOM, with the resultant activation matrix 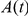, shown on screen (figure 6, panel (c)). The SOM weights matrix 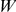 is also displayed (figure 6, panel (a)) to allow the reader to view the unsupervised learning process. The input is a series of samples from a stochastic process in 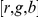. Each colour channel 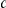 is a scalar updated with:
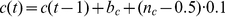
(37)in which 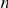 are normally distributed random variables and 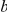 are biases 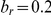, 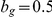, 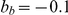. The colour values wrap at 0 and 1. A first-order Markov Model (1MM) is used to predict 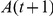 given 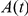. The output of the predictor during the previous iteration 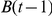 is shown in figure 5, panel (d). Roulette selection from the classification-prediction matrix 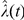 enables the next colour to be predicted and shown. After 3000 iterations of simultaneously training SOM and 1MM, these components are able to classify the current colour and accurately predict the next colour-classification. Euclidean distance between the predicted RGB colour and the next observed colour is 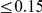. Figure 6, panel (b) shows the current FF input colour and the roulette-selected FB output colour from the previous iteration (these colours should therefore be similar).

### Demo 2: SOM-RSOM Pair

The second demo is simply a reconstruction of the moving-line recognition problem given in Miller et al [35]. The problem will be described only briefly here. The purpose of the problem is to show that a SOM-RSOM pair can perform both spatial and temporal compression, by learning to recognize sequences of visual patterns. In this case, there are 3 sequences to discover:

- No features (blank image)
- A horizontal line that moves from top to bottom of the image
- A vertical line that moves from left to right of the image

**Figure 7:**
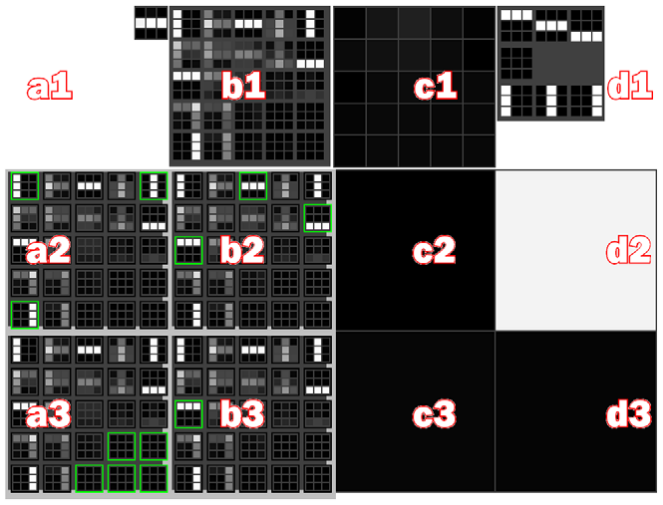
Screenshot, Demo 2. This is a demonstration of a SOM-RSOM pair. A SOM with 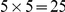 models performs spatial pooling. An RSOM with 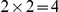 models allows temporal pooling. Panel (a1) shows the hierarchy's FF input, currently mid-way through the horizontal line sequence. (b1) shows the SOM models that have been learnt. (c1) shows the 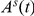, the activation of SOM models given the current FF input. Note that matrices are displayed such that brightness indicates mass or value (i.e. white = 1). (d1) shows the 3 possible input sequences, being pictures of horizontal or vertical lines and a blank image. (a2),(b2),(a3) and (b3) show the four RSOM models. RSOM models represent coincidences of active SOM models and are displayed by outlining significantly (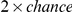) active SOM models in green. After learning, one RSOM model corresponds to horizontal lines (b2) and one to vertical lines (a2). (a3) responds to blank images. Panels (c2),(d2),(c3),(d3) show 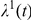, the activity of the RSOM models. The high value in (d2) shows that the RSOM model (b2) is most active, meaning that the hierarchy recognises that it is seeing a moving horizontal line.

The two ‘line’ sequences are interspersed with variable-length sequences of blank images. After 10,000 iterations, the RSOM does indeed develop models that correspond to the two moving line sequences, and a blank-image sequence (figure 7). The SOM has formed models of the visual patterns it receives; the RSOM has associated visual patterns that occur close together in time.

#### Software Interface Details

We will briefly describe the software interface for this demo. A screenshot is included (figure 7). The interface is comprised of a grid of four columns (labelled a,b,c,d) and three rows (1,2,3). Panel a1 shows the FF input to the unit. Panel b1 shows the SOM weights matrix 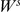. Panel c1 shows the SOM activation matrix 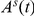. Panel d1 shows the three possible input sequences (horizontal or vertical moving lines and a blank sequence). Panels a2:b3 are arranged in a 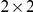 grid and show the state of the RSOM weights matrix 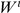. Since each RSOM model represents a distribution of activation of the SOM, we outline SOM models that are significantly active in the corresponding RSOM model. For easy reference, the state of the SOM is shown within each RSOM model. Panels c2:d3 represent 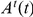, the activation of the grid of 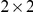 RSOM models. White cells are most active; black cells least active.

### Demo 3: SOM-VMM-RSOM “Words”

**Figure 8:**
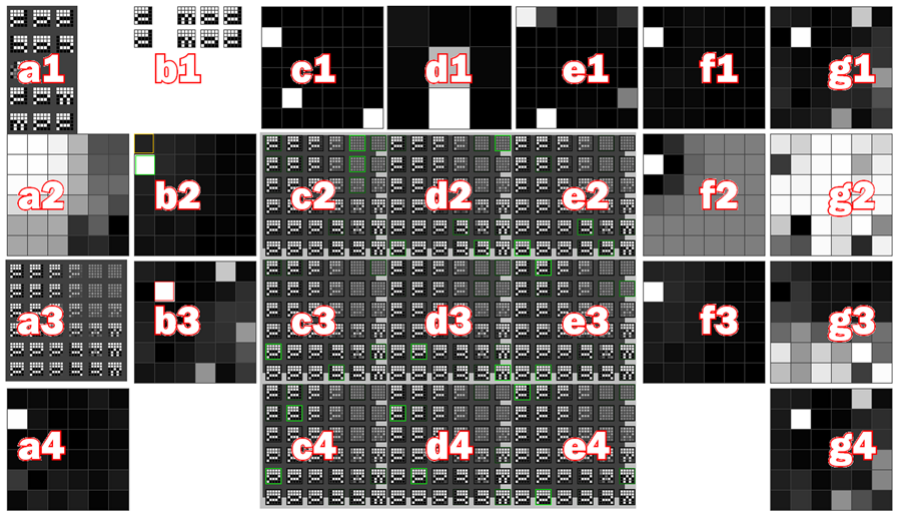
Screenshot, Demo 3. This is a demonstration of first-order versus variable-order prediction. The interface is organised as a 7×4 grid of panels Matrices are displayed such that brightness indicates value (i.e. white = 1). Panel (a1) shows the set of input image sequences (words). (b1) shows the current input ‘d’ and its sequence ‘mad’. The unit's previous predicted letter and sequence are immediately below; these images are simply a copy of the FB output. The unit's SOM has 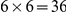 models that are shown in (a3). (a2) shows 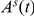, the activation of these models given the current FF input. The RSOM has 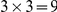 models displayed in panels (c2:e4) by outlining significant SOM models that form each RSOM sequence. The outlines are green, and brightness indicates significance. Note that variable-order bias has created 4 SOM models of ‘a’, representing that letter in different words. The RSOM model in panel (d4) represents ‘mad’. (d1) shows activation of SOM models in response to FF input. Note that the model displayed in (d4) is active. (a4) shows the bias matrix 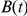. (b2) shows FF SOM classification after biasing, i.e. 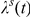. The bias has shifted the best classification from the model outlined in yellow to the model outlined in green, which is the correct ‘d’ for ‘mad’. (b3) shows the FB distribution 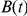 and the roulette-selected model is outlined in red. It predicts all the letters ‘d’, ‘b’, ‘m’ (that start words) and the blank image, because the sequence has finished and the next word is unpredictable. Panels (f1:g4) show internal state of the variable-order predictor including local inhibition (f2) and unpredicted-inhibition (g2).

The objective of this demo is to demonstrate variable-order sequence learning and prediction within the SOM-MPF unit. The problem is inspired by Hawkins et al's paper on sequence memory, [17], in which they describe trying to distinguish the sequences ‘A,C,E’ and ‘B,C,E’. We create a SOM-MPF unit with a variable-order Markov Model (VMM) predictor. We present sequences of noisy images to the unit, each image representing a letter from a word (figure 8). The available words are:

- dad
- bab
- mad
- dam

A random (small) number of blank images are inserted between words. The ordering of words is also random. Eventually, the unit produces RSOM models corresponding to each of the words (and other models corresponding to sequences involving blanks). We measure the ability of the unit to predict each letter. Each iteration, we present the current letter and perform FF and FB passes through the unit. The result of the FB pass is an image of the predicted letter. The normalized distance between the predicted letter and the actual next letter is computed as an error metric.

**Figure 9:**
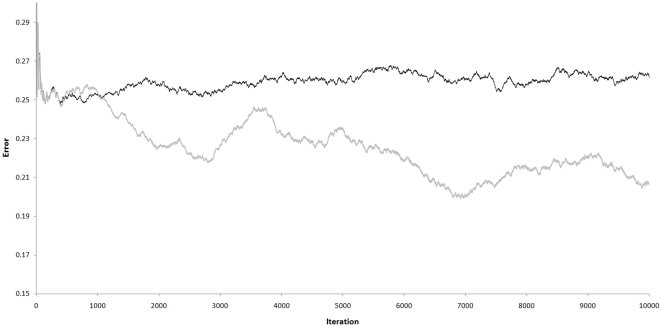
Impact of Variable-Order prediction. These series show prediction error from “Words”, Demo 3. The black series was generated by first-order prediction, the grey series from variable-order prediction. Divergence after 1000 iterations is due to superior predictive ability in the variable-order case. Peaks in the grey series represent periods of “re-modelling” where one or more SOM models are pulled towards participation in two or more sequences, before settling into a local minima. The Y-axis is error, i.e. the Euclidean distance between predicted (FB) and actual (FF) input images. The X-axis shows iterations.

After 10000 iterations, a mean error of 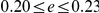 is achieved (averaged over 500 iterations). If a first-order model is used instead, mean error does not decrease below 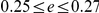 (see figure 9). Error does not reduce to zero because it is impossible to predict that ‘da’ will become ‘dam’ rather than ‘dad’, and because we can't predict when a word will start or which one it will be.

Examination of the SOM models shows that the VMM successfully biases the SOM classification process to produce multiple models corresponding to the same letter, exactly as Hawkins et al anticipated. This also demonstrates the principle for adaptive control, that the FF classification can be deliberately disrupted to achieve secondary objectives without compromising accurate classification (the VMM-biased classification is eventually more accurate than 1MM classification, despite the latter attempting to be as accurate as possible).

The chosen words deliberately include many duplicate letters. By forming separate models for ‘a’, the unit is able to predict that ‘ba’ will not be followed by ‘d’ and ‘ma’ will not be followed by ‘b’. In contrast, the first-order predictor cannot determine which letter will follow ‘a’.

#### Software Interface Details

A screenshot of the software interface for this demo can be seen in (figure 8). The interface is comprised of a grid of seven columns (labelled a,b,…,g) and four rows (1,2,3,4). Matrices are displayed as grids with value 1 being white and 0 as black. Panel a1 shows the five possible input sequences, these being the four words and a random image. Panel b1 shows FF input to the unit (top row) and FB output from the previous iteration (bottom row). The FB output is shown by inverting the SOM and RSOM models to get the expected letter and word respectively.

Panel a2 shows the SOM activation matrix 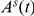. Panel a3 shows the SOM weights matrix 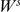. Panel a4 shows 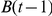, the predicted FF input for the this iteration, generated in the previous iteration. Panel b2 shows 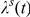, the biased FF output of the SOM. This is the element-wise product of panels a2 and a4. Panel b3 shows 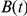, a prediction of the next FF input.

Panel c1 shows previous RSOM FB output, which was used to produce 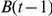. Panel e1 shows current RSOM FB output, which contributed to 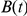. Panel d1 shows RSOM FF output 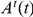. Since in this demo the RSOM is a grid of 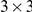 models, 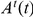 has the same dimension. RSOM weights matrix 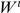 is shown in panels c2:e4. Each RSOM model represents a distribution of activation in the SOM, so to illustrate each RSOM model we display all the SOM models, and outline significantly active models in green.

Panel f1 displays the variable-order predictor (VMM) FF input after preprocessing. Panel g1 shows VMM FF output 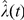 after postprocessing. Panel f2 shows VMM local inhibition around the winning model. Panel f3 shows raw VMM FF input, i.e. 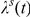 also shown in panel b2. Panel g2 shows inhibition caused by lack of prediction when a strong first-order edge exists. Panel g3 shows local promotion around models inhibited in panel g2. Finally, panel g4 shows VMM FF output before postprocessing.

### Demo 4: “Rocks, Paper, Scissors”

The objective of this demo is to demonstrate that an MPF hierarchy can be used for adaptive control. All control outputs are generated within the hierarchy, not using external learning systems. A pair of units connected via a reward-correlator (RC) form the simplest possible adaptive hierarchy, because the RC needs valid messages to promote or suppress.

“Rocks, paper, scissors” is a game popular with children. Each time it is played, two players simultaneously make one of three gestures representing rocks, paper and scissors. The combination of gestures determines the winner: rock beats scissors, paper beats rock, scissors beats paper, and all other combinations are neutral (a tie). Neither player knows what the other will do, but must guess based on past experience of playing that opponent. In this demo we make an adaptive-MPF hierarchy play against a predictable computer opponent.

The game is played many times. The hierarchy is iterated once every time the game is played. Each iteration consists of a FF and a FB pass of the entire hierarchy. Prior to each iteration, gestures from the previous play are presented to the lower unit 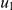 as FF input. After each iteration, the FB output at 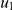 includes both a prediction of the opponent's move, and a move generated by the hierarchy. The latter is compared to the opponent's actual move, and the winner is decided. In this demo, the opponent is restricted to a predictable strategy of cycling through all three gestures in order.

#### Adaptive Components

The reward function for this game is very simple. If the hierarchy won the latest game, 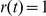. In the case of a tie, 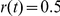. If the hierarchy lost, 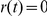. Note that at any iteration 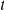 the hierarchy is “perceiving” the previous result and generating the next result.

**Figure 10:**
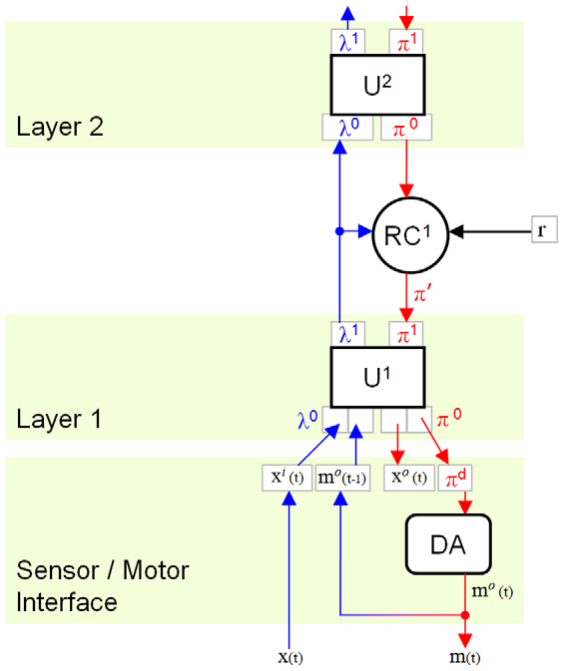
Hierarchy used for demo 4, “Rocks, Paper, Scissors”. Two units are used, with FF and FB messages relayed via a reward correlator (RC). A discrete-actuator component (DA) is used to produce discrete outputs, in this case the 3 gestures.

The sensor-motor interface for this problem is a concatenation of an image and a discrete control output (figure 10). The images are 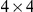 matrices depicting a gesture made by the opponent. The hierarchy observes these gestures. The discrete control output determines the gesture made by the hierarchy. (The hierarchy has the same interface structure both as input to the FF pass and output from the FB pass). However, for effective learning, the hierarchy needs to explore the gamut of possible [sense-act] pairs. This is difficult with discrete motor outputs, because we rely on continuous consequences from small changes in distributions. To ameliorate this problem, a ‘discrete actuator’ (DA) component is added between the lower unit 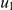 and the motor part of the sensor-motor interface (see figure 10). The DA maps discrete motor actions into probability mass functions over the set of possible actions. Like the SOM-MPF units, the DA has four FF and FB inputs and outputs. The FF input to the DA is a scalar representing the previous output:
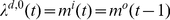
(38)


The FF output of the DA is a PMF over the 3 possible gestures:
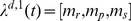
(39)…i.e. 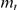 is equal to the probability the move was Rock. Since the move is known, 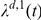 always contains a 1 and two zeros. Since the FB interface vectors are of equal dimension:
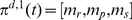
(40)

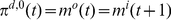
(41)

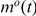
 is selected from 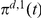 by roulette selection. By concatenating the DA interface with the 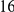 observed gesture pixels 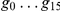, the interface to the hierarchy at 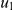 is:
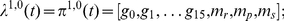
(42)


#### Hierarchy Configuration

Two units are used, with a single reward-correlator (RC) between them (figure 10). The hierarchy is configured without temporal pooling, because we want the reward-correlator to select moves, not sequences of moves. First-order predictors are used in both units, because there are no variable-order patterns to learn. This is the simplest possible configuration of an adaptive-MPF hierarchy, given the stated assumptions about how the hierarchy can be used to produce adaptive behaviour.

Models in the SOM in 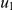 represent the outcome of a single game, including both hierarchy move and opponent's move. The predictor in 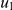 simultaneously predicts both the hierarchy's next move and the opponent's next move. FF messages passed to 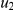 are of the same form, a probability mass function over the SOM models in 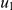. The SOM in 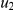 transforms and compresses these distributions; the predictor in 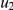 predicts within the transformed and compressed space.

Since there is no higher unit, predictions within 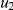 are combined with a uniform distribution during the FB pass of 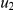. Roulette selection within 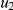 transforms the prediction from 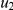 into the a distribution over the SOM models in 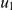. This is relayed back to 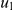 via the reward-correlator, where it is biased towards higher mass for models that are correlated with high reward. In the FB pass of 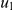 the relayed message is combined with the prediction from 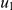. This biases the subsequent FF pass, and is also used to Roulette-select a model from the SOM in 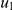 which becomes both a prediction of the next observed gesture, and the probability mass function given to the discrete actuator component. The latter then roulette-selects the move made by the hierarchy.

#### Observations

**Figure 11:**
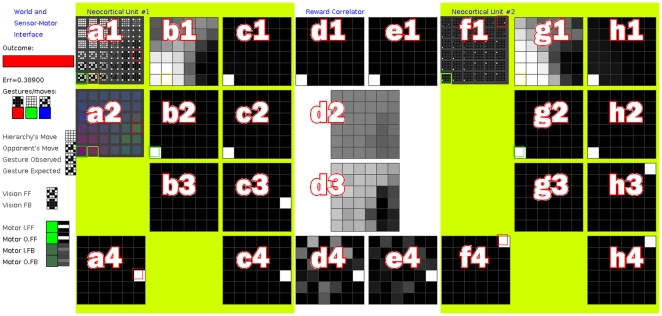
Screenshot, Demo 4. This program shows an adaptive-MPF hierarchy playing “Rocks, Paper, Scissors”. Two units are connected by a reward correlator. 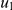
's sensor-motor interface includes observed gestures and motor actions. The latter is a probability mass function (PMF) over the 3 possible gestures. These PMFs can be visualised as RGB values (red is rock, green is paper and blue is scissors). After a little learning, SOM models in 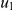 respond to specific gestures but are not specific about the hierarchy's own actions. The motor PMFs are flat (so they appear close to greyscale values). Panel (a1) shows the sensor values of SOM models in 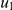. (a2) shows the motor values of SOM models in 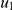. (a4) shows the FB PMF 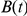 with the roulette-selected action outlined in red. (b1) displays 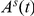, the activation of SOM models in 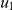. (d1) and (e1) show the FF input and output of the RC. (e4) and (d4) show RC FB input and output respectively. (f1) shows SOM models in 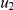 and (g1) shows 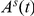 for 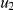. (c2),(c3) show input and output of the first-order predictor in 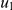 and (h2),(h3) the same for 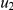.

Within 10000 iterations the SOM in 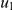 forms recognizable models of the gestures it observes (figure 11). These models also include indeterminate distributions for the agent's own moves. As the neighbourhood of the SOM in 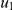 shrinks, these become models of specific combinations of moves from both hierarchy and opponent. The SOM and predictor in 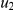 learn more slowly, but are successfully able to represent and predict the sequence of classifications in 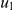. Without a reward-correlator, the hierarchy achieves a mean reward of 0.5, averaged over 500 games. The hierarchy is able to predict its opponent, but has no motivation to do anything about it.

**Figure 12:**
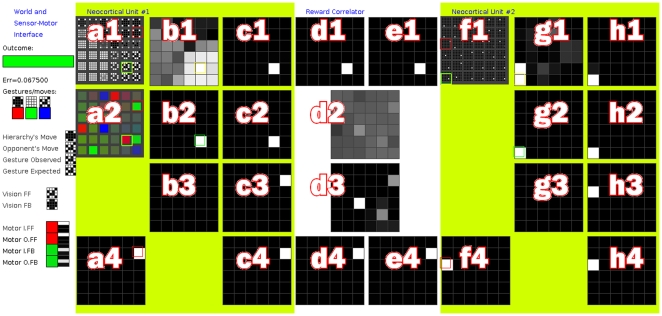
Screenshot, Demo 4. See figure 11 for details of specific panels. After further learning, adaptive pressure from the reward correlator has changed the FB messages between 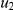 and 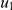 to promote particular SOM models in 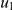. Promoted models represent specific adaptive actions and 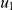 now reliably “predicts” that it will make winning gestures. The hierarchy now wins more than 93% of games it plays.

**Figure 13:**
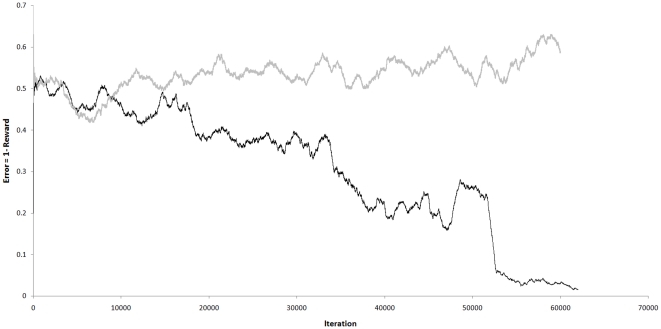
Impact of adaptive bias playing “Rocks, Paper, Scissors”. Values plotted are the inverse of reward averaged over 500 iterations. Reward is maximized and error is zero when the game is won. A draw results in a reward and error of 0.5. The grey series shows outcomes without adaptive bias, a random walk. The black series shows outcomes with adaptive bias, approaching zero (ideal) by 60,000 iterations.

If the messages between units are relayed via a reward-correlator, other changes occur within the units; both predictors “predict” with increasing confidence that the hierarchy will make winning moves (figure 12). A mean reward in the range [0.93,0.98] is reached after 20,000 to 60,000 iterations (figure 13). With random number seed ‘1234’, a mean reward of 0.983 is reached at iteration 61809. The score does not reach 1.0 because a small amount of noise is added to SOM models selected in the FB pass, and 3 Roulette-selections are made before the FB output from 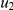 becomes the hierarchy's chosen move. The distributions used for Roulette selection also have a small uniform mass added to them. The hierarchy forms a stable, oscillating system around a maximum reward of 0.93, since losing moves increase the probability of future losing moves, until this trend is reversed by adaptive pressure.

#### Software Interface Details

Two screenshots of the software interface for this demo can be seen in figures 11 and 12. The interface is comprised of a grid of eight columns (labelled a,b,…,h) and four rows (1,2,3,4). Matrices are displayed as grids, with value 1 being white and 0 as black. Since both figures 11 and 12 show the same interface at different times during an experiment, the panels within them are identical. The structure of the hierarchy is two units connected via a reward correlator (RC). Columns a,b,c show the state of unit 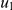. Columns d,e show the state of the RC. Columns f,g,h show the state of unit 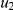.


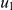
's sensor-motor interface includes observed gestures and motor actions. Observed gestures are small images and can be presented as such. Motor actions are a probability mass function (PMF) over the 3 possible gestures. These PMFs can be visualised as RGB values (red is rock, green is paper and blue is scissors). Panel a1 shows the gesture part of each model in 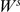 in 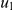. Panel a2 shows the motor part of each model in 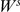 in 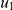. Panel f1 shows 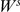 in 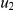, where each model is a distribution of activation of the models in 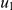.

Panel a4 shows 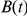 for 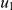 and panel f4 shows 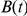 in 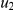. Panels b1 and g1 show SOM activation matrix 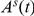 for 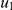 and 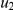 respectively. Panels b2 and g2 show 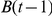, used to bias the current FF output for the two units. Panels b3 and g3 are not in use. Panels c1 and h1 show 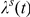. Panels c2 and h2 show first-order Markov predictor (1MM) FF input and panels c3,h3 show 1MM FF output 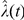. Panels c4 and h4 show the element-wise product of 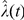 and 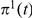, i.e. the bias prior to addition of random noise.

Panel d1 shows the FF input to the RC. Panel e1 shows RC FF output (equal to RC FF input). Panel d2 shows reward correlation matrix 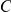. Panel d3 shows adaptive bias. Panel e4 shows RC FB input and panel d4 shows RC FB output, after adaptive bias has been applied.

## Discussion

We have demonstrated that a hierarchical memory system like MPF/HTM can generate adaptive behaviour by exploiting knowledge encoded within and throughout the hierarchy. Since higher layers of the MPF encode concepts with increasing invariances in both space and time, this implies larger hierarchies would generate behaviour using increasingly symbolic or abstract reasoning. The MPF paradigm removes any distinct transition between raw, perceptual data and symbolic representation. Adaptively-biased MPF is a homogeneous system for perception, memory, prediction, planning & control. We believe this direction of research holds much promise in attempts to create an anthropomorphic “general intelligence”.

Although the work described in this paper has some limitations described below, we have met most of our objectives. We have demonstrated that we can balance the conflicting objectives of unsupervised learning of an external world (SOM model learning), and purposeful manipulation of that world via reinforcement learning. The “Reward” used in reinforcement learning can be one or more measurements of anything capable of distinguishing good and bad impacts on the agent. Physical states such as pain, hunger, temperature, exhaustion are all good candidates for reward function[s]. One major benefit of our approach is that when no correlations exist, only transient bias effects are produced (due to noise); therefore, it is possible to include the adaptive biasing technique at all levels within the hierarchy and not require development of specific abstractions/invariances in predetermined layers.

### Constraints and Limitations

The SOM-MPF implementation given in this paper is only an example of the class of algorithms that are described by MPF. Our extensions, particularly adaptive bias, could equally well be added to George et al's HTM algorithm [10]. Use of the Kohonen SOM has some advantages, notably SOM topology allowing implementation of the technique described by Hawkins et al [17] to produce variable-order prediction from first-order models. However, the smoothness of the variation between SOM models also makes it an inefficient technique, if intermediate models are not meaningful. The Recurrent-SOM (RSOM) is not satisfactory for temporal clustering because older classifications become exponentially less significant, making it difficult to represent longer sequences accurately. One solution is to decimate the rate of RSOM update, and smooth its input.

Disrupting hierarchical classification using adaptive bias works, but can be problematic. If the bias is too strong, reality is ignored. If the bias is too weak, the system is not adaptive. Use of a single reward function is difficult but realistic, because the hierarchy should learn to separate the various causes of low reward, such as pain, hunger etc. In our experiment the hierarchy is able to learn that 6 low-reward move-combinations should be avoided. In this paper we rely on temporal pooling to allow actions to be correlated with delayed rewards. A better scheme would be to exploit the discounted future rewards formulation used in reinforcement learning [4].

We chose to correlate states with reward on the FF pass, and modify the messages in the FB pass. This approach is ideal for reward correlation, because FF messages from lower layers are more direct observations of external causes. It is also good for behaviour selection, because FB actions are immediately applied. Other arrangements are possible, such as biasing the FF classification directly.

### Biological Relevance

An interesting question is whether these extensions to MPF are biologically plausible. The answer is beyond the scope of this paper but our extensions have some specific characteristics we can look for.

Existing work on Thalamo-Cortical microcircuits [11] describes messages between hierarchy layers being relayed via the Thalamus. In HTM it is postulated that these messages encode probability distributions over the possible states within each hierarchy node. We proposed to modulate these distributions using a single reward function, to generate adaptive behaviour.

The biological equivalent of our extensions would therefore be a central relay with access to measurements of nonspecific internal states, such as pain or hunger. The relay must be between layers in the cortical hierarchy. Relayed messages would be internally correlated with rewards, and would be adaptively biased (in one or both directions). As a central relay for many cortical areas, it is possible that similar modulation could be part of the role of the Thalamus.

An alternative candidate for our reward-correlating component is the basal ganglia. These are widely believed to have a role in the association of reward and behaviour [38], [39] and there is evidence of circuits connecting the cortex, thalamus and basal ganglia [40], [41]. Interested readers can find models of circuits relating the neocortex and basal ganglia in [42]. Parallels between the function of the basal ganglia and reinforcement learning can be found in [38], [43].

### Future Work

In our next paper we will demonstrate larger hierarchies of 10–20 units successfully playing arcade computer games by screen-scraping and pressing virtual keys. We will also show that by providing an automated commentary in English, the hierarchy is capable of associating words with abstract events in the games. The hierarchy then reproduces the relevant words when executing strategies in the game; in effect, it is able to tell us what it plans to do.

If the MPF is analogous to the human neocortex, then software simulations need to use much bigger hierarchies. The software described in this paper can be used to simulate stable hierarchies of more than 100 units at 30 Hz on a typical laptop computer. We plan to port the code to a massively parallel SIMD platform, to allow realtime simulation of hierarchies of thousands of units. A much larger hierarchy with a high branching factor would have the capacity to combine various derivatives and moments of inputs in many ways, and in consequence the structure of the hierarchy would need less prior design. Eventually we plan to add a detailed vision system and use the adaptive-MPF as the control system for a mobile robot.
